# Energy-Saving Electrospinning with a Concentric Teflon-Core Rod Spinneret to Create Medicated Nanofibers

**DOI:** 10.3390/polym12102421

**Published:** 2020-10-20

**Authors:** Shixiong Kang, Shicong Hou, Xunwei Chen, Deng-Guang Yu, Lin Wang, Xiaoyan Li, Gareth R. Williams

**Affiliations:** 1 School of Materials Science & Engineering, University of Shanghai for Science & Technology, 516 Jungong Road, Shanghai 200093, China; 182442511@st.usst.edu.cn (S.K.); 183762605@st.usst.edu.cn (S.H.); 1826418107@st.usst.edu.cn (X.C.); lixiaoyan@usst.edu.cn (X.L.); 2 Shanghai Institute of Technical Physics, Chinese Academy of Sciences, 500 Yutian Road, Shanghai 200083, China; wanglin@mail.sitp.ac.cn; 3 UCL School of Pharmacy, University College London, 29-39 Brunswick Square, London WC1N 1AX, UK

**Keywords:** energy saving, electrospinning, engineering optimization, poorly water-soluble drug, fast dissolution

## Abstract

Although electrospun nanofibers are expanding their potential commercial applications in various fields, the issue of energy savings, which are important for cost reduction and technological feasibility, has received little attention to date. In this study, a concentric spinneret with a solid Teflon-core rod was developed to implement an energy-saving electrospinning process. Ketoprofen and polyvinylpyrrolidone (PVP) were used as a model of a poorly water-soluble drug and a filament-forming matrix, respectively, to obtain nanofibrous films via traditional tube-based electrospinning and the proposed solid rod-based electrospinning method. The functional performances of the films were compared through in vitro drug dissolution experiments and ex vivo sublingual drug permeation tests. Results demonstrated that both types of nanofibrous films do not significantly differ in terms of medical applications. However, the new process required only 53.9% of the energy consumed by the traditional method. This achievement was realized by the introduction of several engineering improvements based on applied surface modifications, such as a less energy dispersive air-epoxy resin surface of the spinneret, a free liquid guiding without backward capillary force of the Teflon-core rod, and a smaller fluid–Teflon adhesive force. Other non-conductive materials could be explored to develop new spinnerets offering good engineering control and energy savings to obtain low-cost electrospun polymeric nanofibers.

## 1. Introduction

**Figure 1:**
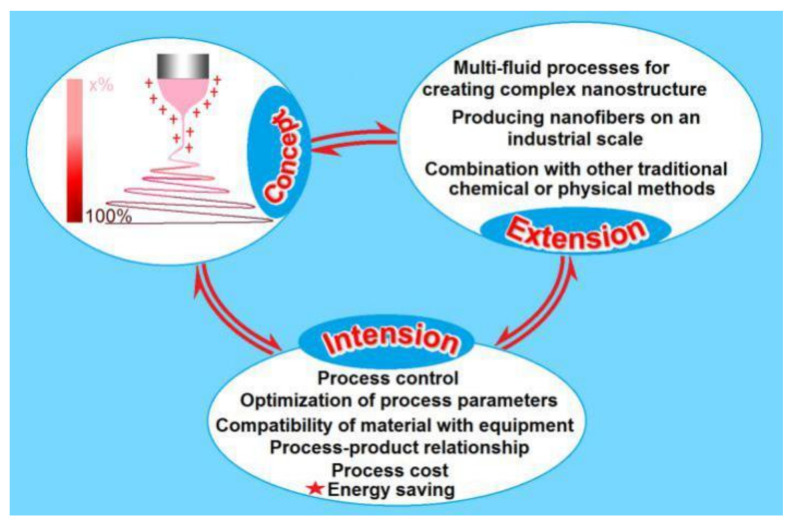
Concept, development, and applications of electrospinning.

Electrospinning, a process in which nanofibers are produced by the interaction of fluids and electrostatic energy [1,2,3,4,5,6], is developing rapidly in two directions. In the first direction, electrospinning is being developed for its capability of generating new types of polymeric nanofibers from different aspects, such as treating more different kinds of working fluids for generating new types of nanofibers [7,8,9,10], implementing the processes in different manners such as coaxial, side-by-side and in combinations [11,12,13], producing nanofibers on a large scale [14,15] and converting the nanofibers through a combination with other traditional chemical or physical methods [14,15,16,17] (Figure 1).

The second direction of developments in electrospinning involves deepening the knowledge about the electrohydrodynamic atomization (EHDA) of working fluids, which is centered around the mechanism of the single-step but “extremely complex” process [18,19,20,21,22]. Most issues in electrospinning have a deep relationship with interface-based engineering considerations [23], such as (1) the contact surface between a fluid and its spinneret that guides the former into the electrical field; (2) the dynamic interface between the working fluid and the environment; (3) fluid–fluid interfacial interactions during a multiple-fluid electrospinning process; and (4) the escape of the solvent from a fluid and the related solidification mechanisms [24]. Although several modified coaxial and triaxial electrospinning processes have been reported based on adjusting the air-fluid contact formats, these processes generally aim to optimize the preparation conditions by employing an additional solvent as a separate working fluid [25,26]. The modified processes have shown great advantages over the traditional processes by enabling the manipulation of the solidification rate of polymeric fluid, which, in turn, generates nanofibers with high quality [27,28]. However, they should inevitably increase the consumption of electrostatic energy, which is neglected in both modified and traditional electrospinning.

Electrical energy can be easily transferred to the working fluid when a metal capillary is exploited as a spinneret, and spinnerets are acknowledged to be the most important part of an electrospinning system. The three other parts of an electrospinning system are the fluid-driving pump, high power supply, and fiber collector [29,30,31]. Thus far, the influence of the surface between the spinneret’s texture and the passing fluid has received little attention. Whereas, the influences are observed in all types of electrospinning methods including, bubble electrospinning, needleless electrospinning, free-surface electrospinning, reactive electrospinning, and the most common needle-based spinneret electrospinning [32,33,34,35,36,37].

Electrospun nanofibers have shown excellent potential applications in many fields, such as the environment, energy, medicine, and functional fabrics [38,39,40]. Many new possibilities in the laboratory may be used to develop new commercial products. Thus, the large-scale production of nanofibers has recently received increased attention [41,42]. Correspondingly, methods to decrease the cost of electrospun nanofibers are of considerable importance. Energy savings during electrospinning will have a considerable influence on the prices of the generated products and, thus, deserve intensive investigation.

In an ideal production run, all of the electrical energy inputted into the electrospinning process is exploited to draw and solidify the working fluids into solid nanofibers. However, some energy is inevitably lost to the environment during the energy transfer and working processes. In an electrospinning system, the spinneret acts as a convergent place of electric energy and the working fluid(s). In the literature, nearly all reported spinnerets are made from metal, especially stainless steel [41,42,43], because metals have fine mechanical properties and are electrically conductive. Thus, this material always comprises spinnerets to transfer electrostatic energy to the working fluid. Whole-metal spinnerets are commonly connected to a power supply through an alligator clip [44,45,46]. However, the metal spinneret and clip could similarly disperse electrostatic energy to the environment through the atmosphere, especially in humid weather. Thus, we hypothesize that other spinnerets consisting of non-metals may be more efficacious in conveying electrostatic energy to the working fluid, which has important implications for large-scale production.

In this study, we developed a concentric spinneret with a solid Teflon-core rod and then used it to implement an energy-saving electrospinning process in which medicated nanofibers consisting of ketoprofen (KET) distributed over a polyvinylpyrrolidone (PVP) matrix were produced. A traditional electrospinning process employing a monoaxial metal tube as a spinneret was also implemented to create nanofibers using the same working fluid. The energy cost of both processes and the properties and performances of the generated nanofibers were compared.

## 2. Materials and Experiments

### 2.1. Materials

Ketoprofen (KET, purity of 98%, white powder) was purchased from Shanghai Hao-Sheng Pharmaceutical Company (Shanghai, China). Polyvinylpyrrolidone K60 (PVP K60, *M*_w_ = 360,000) was obtained from BASF Shanghai Corporation (Shanghai, China). Anhydrous ethanol was provided by Shanghai Shi-Yi Chemicals Reagent Co. Ltd. (Shanghai, China).

### 2.2. Electrospinning Method

The homemade electrospinning apparatus included a power supply (ZGF-60kV/2mA, Wuhan Huatian Co., Ltd., Wuhan, China), two fluid drivers (KDS100 and KDS200 from Cole–Parmer^®^ Corporation, Waltham, MA, USA), a fiber collector (i.e., a hardboard wrapped with aluminum foil), and two homemade spinnerets. One spinneret comprising a 25G stainless steel capillary was utilized to implement a traditional monoaxial process, and the nanofibers obtained from this spinneret were denoted N1. A concentric spinneret with a solid Teflon-core rod was prepared to conduct the energy-saving process, and nanofibers obtained from this spinneret were denoted N2.

The electrospinnable working fluid was prepared by dissolving 10.0 g KET and 40.0 g PVP K60 in 500 mL anhydrous ethanol; the final concentrations of KET and PVP were 2% (*w*/*v*) and 8% (*w*/*v*), respectively. The fiber collection distance and fluid flow rate were fixed at 20 cm and 12 kV, respectively. A drop shape analysis instrument (DSA100, Kruss GmbH, Hamburg, Germany) was exploited to measure contact angles of working fluid and the metal and Teflon surfaces.

### 2.3. Characterization

N1 and N2 were assessed using a scanning electron microscope (S-4800, Hitachi, Japan). The diameters of the nanofibers were estimated from over 100 points on the SEM images using ImageJ software (NIH, Bethesda, MD, USA). The samples were gold-sputtered for 90 s. The physical status of KET and PVP in N1 and N2 was investigated using an X-ray diffractometer (Bruker-AXS, Karlsruhe, Germany) within the 2θ range of 5–60°. The samples were also subjected to Fourier transform infrared spectrometry (FTIR; Spectrum 100 Perkin–Elmer, Billerica, MA, USA) in the range of 500–4000 cm^−1^.

### 2.4. Functional Performance

#### 2.4.1. Drug Encapsulation Efficiency

Quantitative measurements of KET were conducted using a UV-vis spectrophotometer (Unico Instrument Co., Ltd., Shanghai, China). The absorbance of the KET solution at λ_max_ = 260 nm was exploited to build a standard equation from standard solutions and calculate the KET concentration of samples of unknown concentrations.

The nanofiber mats were cut into 2 cm^2^ pieces at random locations and weighed to determine the KET contents of N1 and N2. The samples were placed in separate flasks containing 100 mL of distilled water and stirred at 500 rpm continuously for 30 min. The absorbance of the solution was measured, and the KET concentration was calculated (PVP has no absorbance at 260 nm). The encapsulation efficiency (EE) of KET was calculated using Equation (1) [47]:

(1) $$EE(\%)=\frac{C_{d}}{C_{t}}\times 100\%$$

where *C_d_* is the KET content determined from the electrospun nanofibers by experimentation and *C_t_* represents the theoretic KET concentration according to the preparation conditions, i.e., 20% (*w*/*v*).

#### 2.4.2. In Vitro Dissolution Tests

A paddle method according to the Chinese Pharmacopeia (2015 ED) was exploited to measure the drug release profiles of N1 and N2 during in vitro dissolution tests. A dissolution apparatus (RCZ-8A, Tian-Jin University Radio Factory, Tian Jin, China) with seven vessels was utilized. Each vessel was filled with 900 mL of phosphate buffered solution (PBS, pH 7.0, 1.0 M) at 35 ± 1 °C and 50 rpm. N1 or N2 (0.2 g × 6 pieces) was placed in six of the seven vessels. At selected time points, 5.0 mL of the aqueous solution was withdrawn and 5.0 mL of fresh PBS from the seventh vessel was added to maintain a constant volume of the dissolution medium.

The cumulative percentage release of KET (*Q*) was calculated according to Equation (2) [48]:

(2) $$Q\%=\frac{C_{n}\times V_{0}+\sum_{i=1}^{n-1}C_{i}\times V}{C_{0}V_{0}}\times 100\%$$

where *V*_0_ and *V* represent the volumes of the bulk dissolution medium (900 mL) and withdrawn sample (5 mL), respectively, *C*_0_ represents the theoretical concentration of KET after complete release, *C*_n_ represents the measured KET concentration determined in the *n*th aliquot (μg/mL), and *C*_i_ is the KET concentration in the *i*th aliquot (μg/mL).

#### 2.4.3. Ex vivo Drug Permeation Tests

Ex vivo permeation tests of KET from N1 and N2 and raw KET powders were implemented by using a permeation test apparatus (RYJ-6A, Shanghai Huang-Hai Drug Control Instrument, Shanghai, China) This apparatus contained six Keshary–Chien glass diffusion cells and a water-bath system to maintain a constant temperature (35.0 ± 0.5 °C). The samples were mounted in the cells, which had a diffusion area of 2.60 cm^2^. The receptor compartment had a volume of 7.2 mL.

Porcine samples of sublingual mucosa were obtained from a local abattoir and mounted between the receptor and donor compartments of the diffusion cells; here, the mucosal surface faced upward. Each donor compartment was filled with 1.0 mL of PBS. A magnetic bead was placed in the receptor compartment and stirred at 100 rpm. Prior to the permeation tests, the sublingual membranes were equilibrated for 30 min. N1 or N2 (10 mg) or 2.0 mg of raw KET powder (<0.1 mm) was placed on the mucosal surface. Exactly 1.0 mL of each sample was withdrawn from the receptor at selected time points and replaced with 1.0 mL of fresh PBS. The withdrawn samples were filtered through a 0.22 µm membrane (Millipore) for KET concentration measurements. The cumulative permeation rate of KET (*P*) was calculated according to Equation (3) [49]:

(3) $$P\%=\frac{C_{n}\times V_{0}+\sum_{i=1}^{n-1}C_{i}\times V}{Q_{\infty }}\times 100\%$$

where *V_0_* and *V* represent the volumes of the bulk dissolution medium (7.2 mL) and withdrawn sample (1.0 mL), respectively, *C*_n_ represents the KET concentration determined in the nth aliquot (μg/mL) from the receptor compartments, *C_i_* is the KET concentration in the *i*th aliquot (μg/mL), and *Q_∞_* represents the KET content (μg) placed in the donor compartments.

### 2.5. Statistical Analysis

All experiments were conducted at least three times, and all values are expressed as mean ± standard deviation. A *p* value of <0.05 indicated a statistically significant difference.

## 3. Results and Discussion

### 3.1. Designs of the Concentric Spinneret with a Teflon-Core Rod

**Figure 2:**
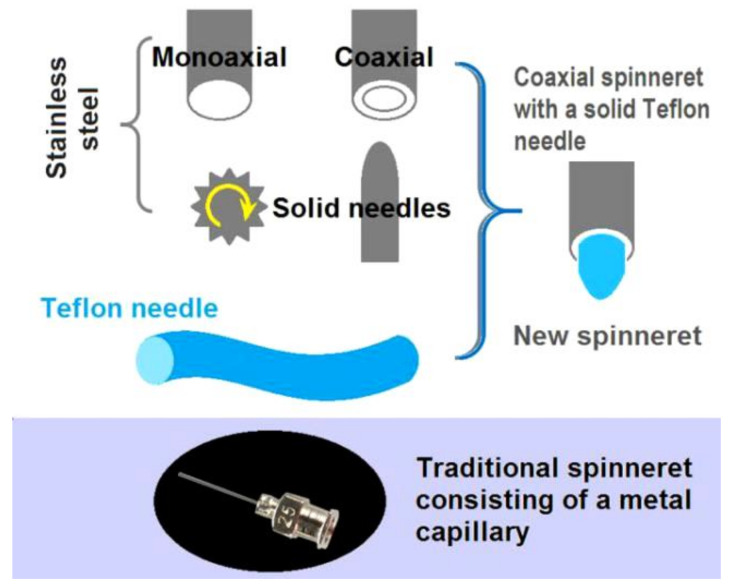
Conceptualization of the concentric spinneret with a solid Teflon-core rod and a traditional stainless steel needle utilized as the control.

The original idea behind the concentric spinneret with a solid Teflon-core rod is shown in Figure 2; a traditional stainless steel needle utilized as the control is also illustrated. The homemade spinneret combines the features of a traditional monoaxial spinneret, a coaxial spinneret, and a solid needle spinneret. The tube-based spinneret could guide the fluid in the electrical field with greater accuracy than needless electrospinning. The solid needle-spinneret has the advantage of a free surface, which could overcome negative capillary forces [50,51]. Thus, this combination should have the advantages of both single-fluid processes with solid needle or tube needle, and is organized in a concentric manner for convenient implementation.

The concentric spinneret is made of stainless steel, epoxy resin, and a Teflon rod as the core. The metal tube presents good conductivity for energy transfer and strong mechanical properties to enable facile installation of the electrospinning system but could divert energy toward random directions and easily interact with the viscous fluids. The Teflon rod has the advantage of a low surface energy for rejecting clinging and dielectric, which means most of the energy could be concentrated on the fluids. The epoxy resin can prevent energy dispersion to the environment. The traditional spinneret, which is shown at the bottom of Figure 2, consists of a metal capillary of 25G, has inner and outer diameters of 0.26 and 0.51 mm, respectively, and is used to carry out tube-based single-fluid electrospinning.

**Figure 3:**
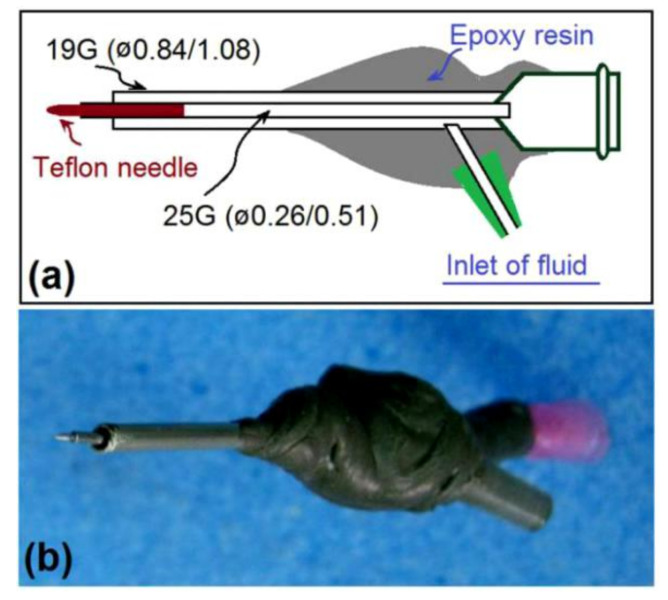
Proposed concentric spinneret with a solid Teflon rod. (**a**) Diagram showing the inner structure of the spinneret. (**b**) Digital photograph of the complete spinneret.

A schematic of the preparation process and a digital photograph of the new spinneret are shown in Figure 3a,b, respectively. The spinneret was modified from a traditional concentric spinneret; here, a 25G metal capillary was nested into a 19G tube with inner and outer diameters of 0.84 and 1.08 mm, respectively. A Teflon rod was sharpened and inserted directly into the inner tube to form the Teflon-core rod. Epoxy resin was utilized to seal joints and cover all metal surfaces except for a short section through which the alligator clip could convert electrostatic energy.

### 3.2. Implementation of the New Electrospinning Process

**Figure 4:**
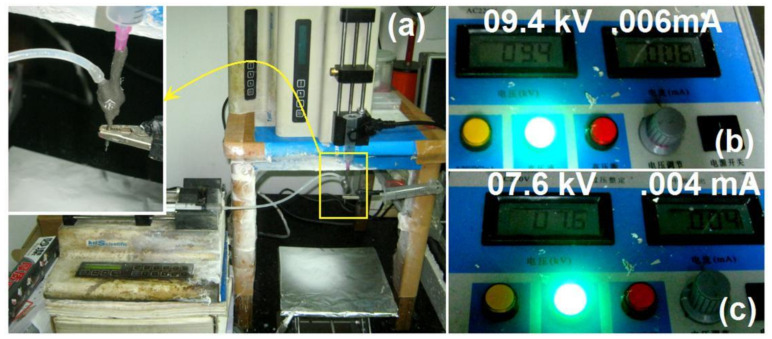
Comparison of the energy consumption of electrospinning processes using the traditional metal tube-based spinneret and the new concentric spinneret. (**a**) Digital photograph of the working process. Top-left inset: Connection of the small naked metal surface for electrostatic energy transfer. (**b**) Applied voltage and current of the traditional process. (**c**) Applied voltage and current of the new process.

The groove connected to the core tube and solid Teflon needle could be inserted with an empty plastic syringe and directly fixed to a pump. The working fluid could be pumped quantitatively into the spinneret through an elastic silicone tube, as shown in the top-left corner of Figure 4a. The Chinese character on the epoxy resin in the spinneret was utilized to facilitate good focus when photographs of the working process were taken under a certain magnification using a camera.

When the conventional single-fluid process was conducted using the 25G spinneret, a high voltage of 9.4 kV was necessary to maintain a continuous and robust working process and the responding current was 0.06 mA (Figure 4b). When the new spinneret was utilized to implement electrospinning under the same fluid flow rate of 2.0 mL/h, the applied voltage was 7.6 kV and the current reported by the high-voltage generator was 0.04 mA (Figure 4c). The whole-metal spinneret, although convenient for electrospinning operations, requires a higher voltage and larger current for the working process. The differences should be resulted from the energy lost to the environment when compared with the new spinneret, although it should also lose some energy to the surroundings.

**Figure 5:**
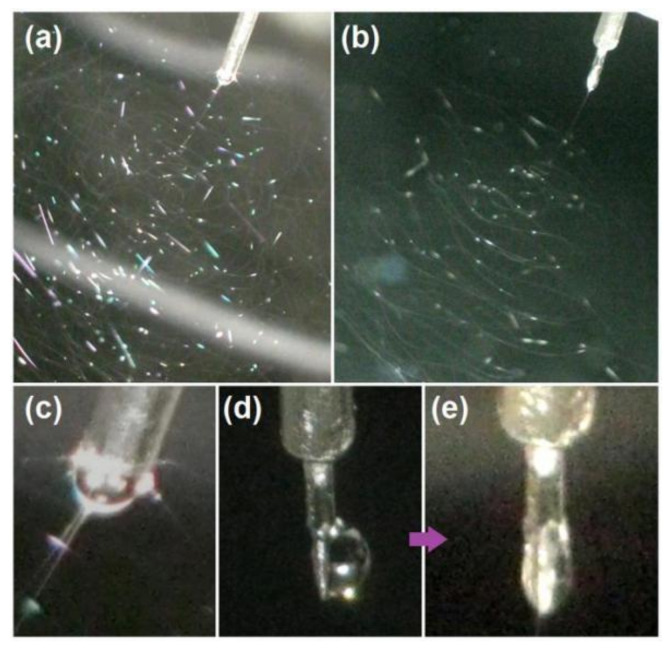
Working process. (**a**) Digital photograph of the traditional working process based on a metal capillary as the spinneret. (**b**) Digital photograph of the working process based on the concentric spinneret with solid Teflon rod. (**c**) Digital Taylor cone. (**d**,**e**) Change in the liquid droplet to Taylor cone on the Teflon tip under an applied voltage of 7.6 kV.

Digital photographs of the working process using the two spinnerets are shown in Figure 5. Figure 5a,b reflects traditional tube-based and the new Teflon rod concentric spinneret-based processes, respectively. Similarly, both of them had the typical three successive steps for finishing the solidification of working fluid to solid nanofibers, i.e., Taylor cone, straight fluid jet and the unstable regions.

Considerable differences may be observed when the two working processes are compared in detail. First, the Taylor cones of the two processes are different. The Taylor cone of traditional single-fluid tube-based electrospinning is shown in Figure 5c; the Taylor cone obtained reflects a common liquid cone with arched air–fluid boundaries hanging at the tip of the tube’s outlet nozzle. Shown in Figure 5d is a standing liquid of the working fluid on the Teflon surface due to its hydrophobic property before the voltage was applied. When the applied voltage was increased to 7.6 kV, a typical stable Taylor cone was obtained, as shown in Figure 5e. The cone had a “big in the middle but small at the two ends” image, which could be the systematic result of actions from the “air-fluid” free surface and “fluid-Teflon surface”. The tip of the Taylor cone is almost the tip of the Teflon rod in Figure 5e, thus suggesting easy initiation of the electrospinning process due to the low surface energy between the fluids and Teflon surface and the absence of negative capillary forces that draw the fluid back into the tube. The lengths of the straight fluid jet for the new and tube-based processes are 3.2 and 2.4 mm, respectively (estimated with the spinneret as a reference in Figure 5a,b). Additionally, the bending and whipping loops in Figure 5b are looser than those in Figure 5a, thus suggesting the presence of large repelling forces between the two adjacent loops. These phenomena reveal that more electrical energy is converted into the working fluids during electrospinning with a concentric spinneret with a Teflon-core rod than during electrospinning with a traditional spinneret.

### 3.3. Energy-Saving Effect and the Related Micro-Formation Mechanism

The power consumption (*P*, W) of the electrospinning process can be calculated according to Equation (4):

*P* = *U × I* (4)

where *U* and *I* represent the applied direct voltage and electric current, respectively. For tube-based electrospinning, *W*_t_ = *U × I* = 9400 × 0.06 × 10^−3^ = 0.564 W. For new spinneret-based electrospinning, *W*_n_ = *U × I* = 7600 × 0.04 × 10^−3^ = 0.304 W. Thus, the new electrospinning process requires only 0.304/0.564 × 100% = 53.9% of the energy consumed by the traditional electrospinning process. This result suggests that the proposed spinneret promotes an effective energy-saving working process.

**Figure 6:**
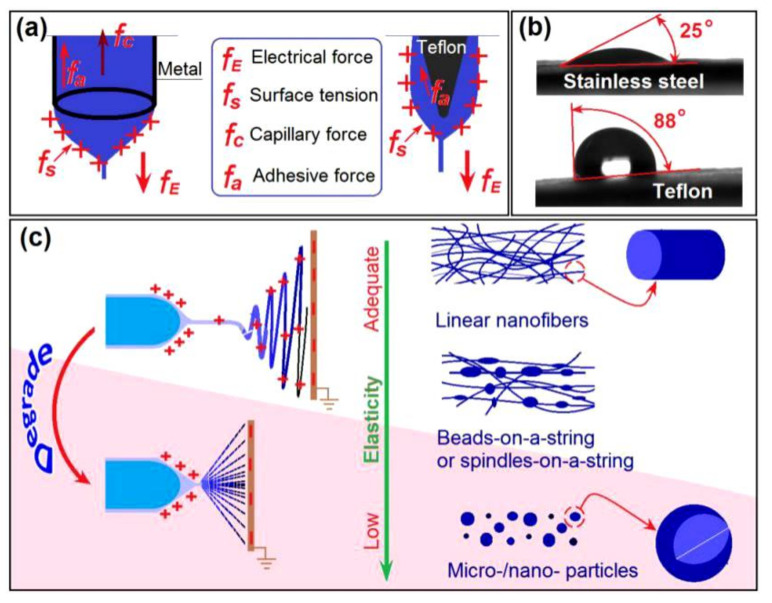
Mechanism of energy saving based on the concentric spinneret with a Teflon-core rod and the micro-formation of nano products. (**a**) Backward forces that waste electrical energy. (**b**) WCA showing adhesive forces between the working fluids on different surface textures. (**c**) Changes in products as a function of the PVP concentration in the fluids.

Compared with capillary-based electrospinning, the energy-saving effect of the new process can be summarized as follows: (1) Most of the surface of the spinneret is covered by the insulating polymers including epoxy and PP plastic exception a section of the metal tube for the alligator clip, by which the electric energy dispersing to the environment is blocked. (2) The Teflon-core needle, as an insulating material, retains the electrical energy in the working fluid when it is guided into the electrical field. (3) Diagrams of the forces exerted on the Taylor cone of the tube-based and proposed electrospinning processes are shown in Figure 6a. In the former process, the electrical force *f*_E_ must overcome the surface tension *f*_s_, capillary force *f*_c_, and adhesive force *f*_a_. By contrast, the new spinneret has no *f*_c_ and *f*_a_ is smaller than that of the metal surface. This can be determined from the spreading angle when 3 μL of the working fluid was put on the surface of stainless steel and Teflon, a smaller contact angle (after 5 s) of 25 ± 4° than 88 ± 7° suggests better compatibility and a larger *f*_a_ on the metal surface (Figure 6b). The presence of *f*_c_ and *f*_a_ mean unnecessary energy loss; thus, the new process can reduce energy loss during the working process. In other words, the new spinneret can avoid energy loss during the electrospinning process through the exploitation of insulating polymeric materials for coverage, the solid core needle, the free-surface fluid leading format, and the low fluid-Teflon surface adhesion.

The key element for forming nanofibers with a fine linear morphology is the property of the working fluid. Similar to that in tube-based electrospinning, a working fluid with the appropriate apparent viscosity (or essentially enough physical entanglements of polymeric chains) would result in sufficient resistant forces to balance the electrical drawing forces, through which the liquid fluids are solidified into solid nanofibers. If the working fluid does not have enough viscosity, the products could degrade into beads-on-a-string, spindles-on-a-string morphologies, or even micro- or nanoparticles. Given variations in the PVP and KET concentrations in the working fluids, products in the forms of nanofibers, beads-on-a-string, and solid particles could also be generated using the new concentric spinneret with a Teflon-core rod (Figure S1 and Table S1 in the Supplementary Materials). Thus, the present strategy could be explored to conduct other EHDA processes with considerable energy savings.

### 3.4. Properties, Physical State, and Compatibility of the Nanofibers

**Figure 7:**
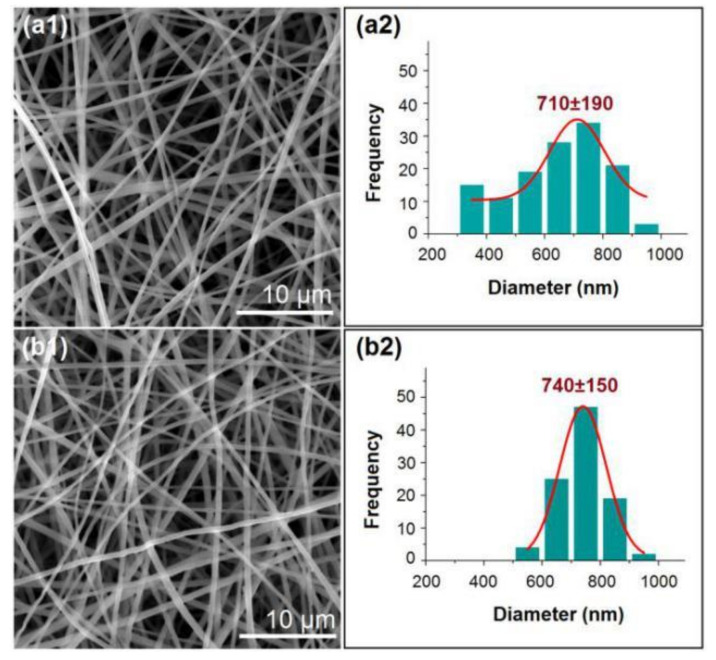
SEM images and size distributions of (**a1**,**a2**) nanofibers from the traditional processes (N1) and (**b1**,**b2**) nanofibers from the new process (N2).

SEM images of N1 (Figure 7a1) and N2 (Figure 7b1) reveal linear morphologies without the spindles-on-a-string phenomenon. The average diameters of N1 and N2 are 710 ± 190 nm (Figure 7a2) and 740 ± 150 nm (Figure 7b2), respectively. The electrospinnability of the co-dissolving working fluid indicates that these nanofibers are similarly straight despite being produced under different systems and applied voltages.

**Figure 8:**
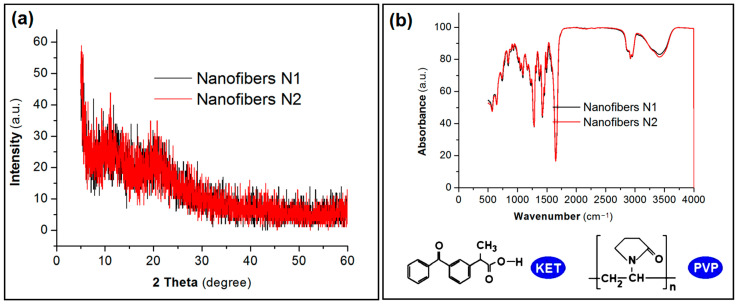
XRD patterns (**a**) and ATR-FTIR spectra (**b**) of nanofibers N1 and N2.

The XRD curves of N1 and N2 are shown in Figure 8a. No sharp peaks corresponding to Bragg reflections could be discerned, which means KET is present in the nanofibers in an amorphous state regardless of the latter’s production method. The FTIR spectra of N1 and N2 are also similar, thus suggesting that they are similar drug–polymer composites (Figure 8b). Each KET molecule has a –OH group, while PVP molecules have many –C=O groups. Thus, hydrogen bonds can be formed between these groups so that KET molecules fill the voids among the entanglements of PVP molecules. This space separation effect would be useful in retarding the recrystallization of KET molecules into dimers or new crystals.

### 3.5. Functional Performances of the Fabricated Nanofibers

The KET contents *C_d_* measured from N1 and N2 are 19.62 ± 1.27% (w/w) and 19.49 ± 1.41% (w/w), respectively. The similar encapsulation efficiency values of *EE%* (i.e., 98.1 ± 6.4% and 97.5 ± 7.1% for N1 and N2, respectively) suggest that the electrospinning is essentially a physical drying process without any drug loss to the environment, although reactive electrospinning has been reported in literature [52,53,54]. Drying at an extremely rapid rate should be a unique advantage of electrospinning over many other bottom-up methods used for creating nanomedicines [55,56].

**Figure 9:**
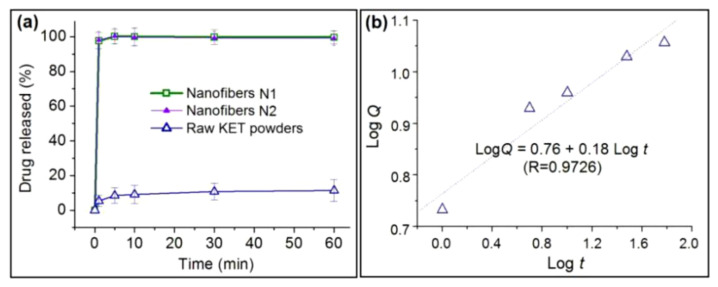
In vitro dissolution profiles of N1, N2, and raw KET powders (**a**), and the drug release mechanism of KET powder (**b**).

Comparisons of the drug dissolution performances of N1, N2, and the raw KET powders are shown in Figure 9a. N1 and N2 respectively released 97.7% ± 4.7% and 98.1% ± 5.1% of their loaded KET within the first minute of dissolution, thereby suggesting an immediate drug release effect. By contrast, the raw KET powders released only 5.4% ± 3.2% of the added KET powders; this rate is nearly 20 times slower than those of the composite nanofibers.

Drug release mechanisms are traditionally analyzed using the Korsmeyer–Peppas Equation (5) [57,58]:

(5) $$LogQ=Log\frac{M_{t}}{M_{0}}=Logk+nLogt$$

where *Q* is the release percentage, *M*_t_ and *M*_0_ represent the drug release at time *t* and the total amount of the loaded drug, *k* is a kinetic constant, and *n* is a coefficient indicating the drug release mechanism. Here, *n* ≤ 0.5 represents a Fickian diffusion mechanism, *n* ≥ 1 represents an erosion mechanism, and 0.5 < *n* < 1 represents a combined mechanism of erosion and diffusion. In the present studies, N1 and N2 exhausted all of the loaded KET within 1 min, with not enough data to regress the equations. However, the drug is released by an erosion mechanism, i.e., KET molecules hanging on the PVP molecules are dissolved with the dissolution of PVP molecules, which could be easily deduced. The regression equation for the KET powders is *L*og*Q* = 0.76 + 0.18*L*og*t* (*R* = 0.9726). An *n* value of 0.18 indicates that KET dissolves from its crystalline powders through a typical Fickian mechanism. By comparison, the mechanisms of N1 and N2 have revised the drug dissolved behaviors and release mechanisms, from a gradually surface diffusion to an immediate erosion.

**Figure 10:**
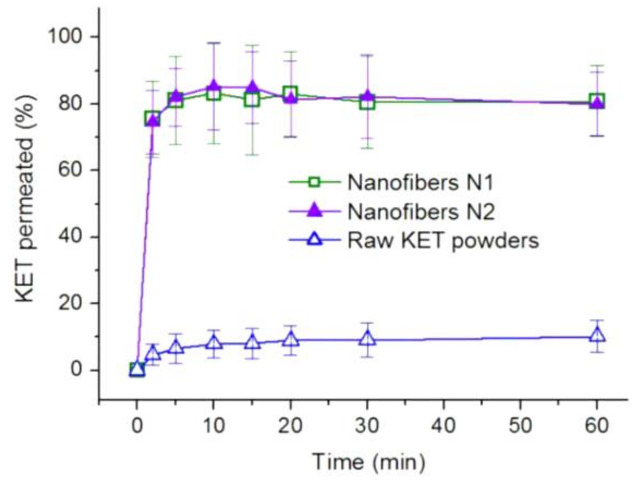
Ex vivo permeation tests of N1, N2, and the raw KET powders.

The ex vivo permeation profiles of KET from N1, N2, and the raw KET powders are illustrated in Figure 10. After 2 min (the first time point), the cumulative permeation percentages of N1 and N2 were 75.47% ± 11.4% and 74.65% ± 9.6%, respectively. After 10 min, the peak permeation percentages of KET in N1 and N2 reached 83.17% ± 15.1% and 85.24% ± 13.3%, respectively. These permeation data suggest no significant difference between the nanofibers. By comparison, after 10 min, the permeation percentage for the raw KET powders was 7.89% ± 4.22% Thus, N1 and N2 provide permeation rates over 10 times faster compared with that of the KET powders for rapid drug delivery. KET is a typical class II nonsteroidal anti-inflammatory drug with a poor solubility. The dissolution rate of this drug is the key factor limiting its delivery to the circulation system. The results indicate that N1 and N2 could significantly improve the dissolution speed and promote the trans-membrane rate of KET for fast delivery and immediate therapeutic action.

**Figure 11:**
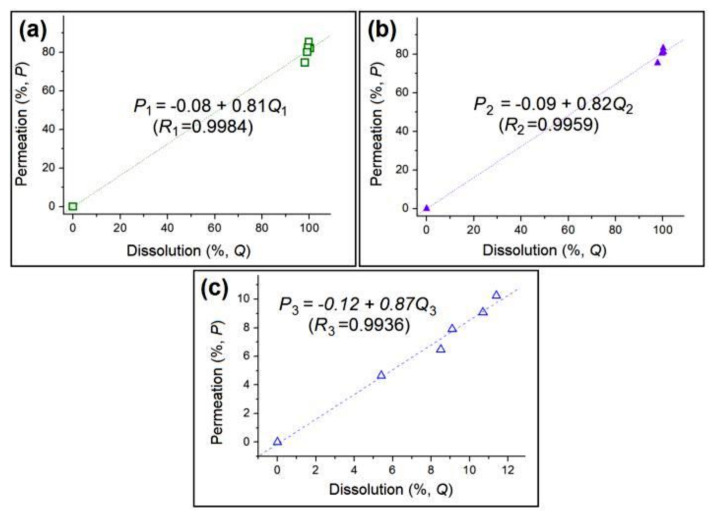
Relationship between the in vitro dissolution and ex vivo permeation data of N1 (**a**), N2 (**b**), and the raw KET powders (**c**).

Therapeutic concentrations of a poorly water-soluble drug in the circulatory system are achieved not only by fast dissolution after oral administration but also by rapid permeation of the drug through bio-membranes [59,60]. KET has good compatibility with bio-membranes [61]. Correlations of the drug in vitro dissolution data with the permeation data are shown in Figure 11. The regression equations for N1, N2, and raw KET powders are *P*_1_ = −0.08 + 0.81*Q*_1_, *P*_2_ = −0.09 + 0.82*Q*_2_, and *P*_3_ = −0.12 + 0.87*Q*_3_, respectively, and the correlation coefficients of these equations are 0.9984, 0.9959, and 0.9936, respectively. These correlation coefficients suggest that the permeation rates have a close relationship with the dissolution rates. Thus, the dissolution of KET may be expected to be a dominant step for its therapeutic action. N1 and N2 are able to release the loaded KET molecules nearly 20 times faster than the raw KET powders and show 10 times faster permeation rates than KET powders to induce rapid therapeutic effects. Oral dispersible films are among the most favored dosage forms of patients [62,63]. The unique properties of electrospun nanofibers reflect their potential applicability to oral dispersible films with great commercial value [64,65]. The reported KET-PVP films immediately dissolve when they encounter water (Figures S2 and S3 in the Supplementary Information) and may be fine candidates as dispersible films for fast trans-membrane drug delivery.

Although nanofibers N1 and N2 show no significant differences in terms of their properties and functional performances for rapid drug delivery, they have completely different energy consumption. A high voltage must be delivered to the working fluid to initiate the electrospinning process. However, methods to improve the effectiveness of the delivery process to charge the working fluids are scarce. The fluid charging process could be divided into two steps, and each step could be optimized by various engineering methods. The first step involves the transfer of electrical energy to the working fluid in an efficacious manner without loss to the environment, which can be fulfilled by an insulated polymer-covered surface on the spinneret. Here, an epoxy resin coverage is provided; the metal material easily converts the energy to the fluid but also disperses the energy to the atmosphere from all directions. The second step involves the initiation of the Taylor cone and the effective ejection of the straight fluid, which could be realized by a free-surface process with low surface adhesion. Here, the Teflon-core needle is utilized. The latter energy–fluid interactions in the unstable region and straight fluid jet are self-working procedures that are mainly influenced by the properties of the working fluids. The applications of electrospun nanofibers as commercial products need to resolve two important issues, i.e., production on a large scale and at a relatively low cost [66,67]. In the literature, many new methods have been reported about the creation of nanofibers on a large scale such as multiple-needle electrospinning [68], needleless electrospinning [69], multinozzle air-jet electrospinning [70], and so on. The strategy reported here for cutting the cost of electrospun nanofibers due to electrical consumption can be combined with those large-scale production methods for a bright future of commercial electrospun products.

## 4. Conclusions

Two key steps influence the efficiency of electric energy to drive electrospinning, i.e., the effective delivery of a high voltage to the working fluid and the easy initiation of the Taylor cone. Two engineering strategies were explored to implement an energy-saving process based on a homemade spinneret with a concentric structure but a solid Teflon-core needle. Compared with traditional metal tube-based electrospinning, the new process consumed only 53.9% of the electric energy for a similar preparation. The nanofibers from the two processes showed no significant differences in terms of morphology, size distribution, and amorphous properties. The nanofibers had similar in vitro dissolution rates and ex vivo drug permeation functional performances, which were over 10 times better than that of raw KET powders. As commercial electrospun products are increasingly introduced to the market, energy savings in working processes could be expected to draw attention in efforts to reduce the production cost of functional nanofibers. The proposed protocol presents a new electrospinning technique that could be employed for energy-saving nano fabrication and explorations of new theoretical issues associated with electric energy–fluid interactions in EHDA.

## Supplementary Materials

The following are available online at https://www.mdpi.com/2073-4360/12/10/2421/s1, Figure S1: Polarized microscopic images of the EHDA products from working fluids with varied PVP and KET concentrations, which were created using the coaxial spinneret with a core solid Teflon rod: (a) E1; (b) E2; (c) N2; (d) E3; (e) E4; (f) E5. Figure S2: Observations of the fast disappearance of a drop of water when it is placed on the surface of electrospun nanofibers N1. Fig. S3. Observations of the fast disappearance of a drop of water when it is placed on the surface of electrospun nanofibers N2. Table S1: The parameters for EHDA processes on different working fluids.

## References

[1] An S., Kim Y.I., Jo H.S., Kim M.-W., Lee M.W., Yarin A.L., Yoon S.S. (2017). Silver-decorated and palladium-coated copper-electroplated fibers derived from electrospun polymer nanofibers. Chem. Eng. J..

[2] Kowalczyk T. (2020). Functional micro- and nanofibers obtained by nonwoven post-modification. Polymers.

[3] Wang M., Hou J., Yu D.-G., Li S., Zhu J., Chen Z. (2020). Electrospun tri-layer nanodepots for sustained release of acyclovir. J. Alloys Compd..

[4] Marilena V., Stefanos K., Angeliki S., Konstantina T., Stefania K., Efstathia I., Vassilios R., Andrew T. (2019). Fabrication and characterization of electrospun nanofibers for the modified release of the chronobiotic hormone melatonin. Curr. Drug Deliv..

[5] Said S.S., Campbell S., Hoare T. (2019). Externally addressable smart drug delivery vehicles: Current technologies and future directions. Chem. Mater..

[6] Minden-Birkenmaier B.A., Smith R.A., Radic M.Z., van der Merwe M., Bowlin G.L. (2020). Manuka honey reduces NETosis on an electrospun template within a therapeutic window. Polymers.

[7] Nagy Z.K., Wagner I., Suhajda A., Tobak T., Harasztos A.H., Vigh T., Soti P.L., Pataki H., Molnar K., Marosi G. (2014). Nanofibrous solid dosage form of living bacteria prepared by electrospinning. Express Polym. Lett..

[8] Smrithi P., Deepthy M. (2019). Nanofibrous polydioxanone depots for prolonged intraperitoneal paclitaxel delivery. Curr. Drug Deliv..

[9] An S., Lee M.W., Yarin A.L., Yoon S.S. (2018). A review on corrosion-protective extrinsic self-healing: Comparison of microcapsule-based systems and those based on core-shell vascular networks. Chem. Eng. J..

[10] Aytac Z., Ipek S., Erol I., Durgun E., Uyar T. (2019). Fast-dissolving electrospun gelatin nanofibers encapsulating ciprofloxacin/cyclodextrin inclusion complex. Colloids Surf. B..

[11] Kazsoki A., Farkas A., Balogh-Weiser D., Mancuso E., Sharma P.K., Lamprou D.A., Zelkó R. (2020). Novel combination of non-invasive morphological and solid-state characterisation of drug-loaded core-shell electrospun fibres. Int. J. Pharm..

[12] Wang M., Li D., Li J., Li S., Chen Z., Yu D.-G., Liu Z., Guo J.Z. (2020). Electrospun Janus zein–PVP nanofibers provide a two-stage controlled release of poorly water-soluble drugs. Mater. Design.

[13] Liu Y., Liu X., Liu P., Chen X., Yu D.G. (2020). Electrospun multiple-chamber nanostructure and its potential self-healing applications. Polymers.

[14] Liu Z., Zhao J., Zhou L. (2019). Recent Progress of the needleless Electrospinning for high throughput of nanofibers. Recent Pat. Nanotechnol..

[15] Nawzat D.A.L.J., Mohammad D.B., Jolius G., Alam A.K.M.M. (2019). An overview of chitosan nanofibers and their applications in the drug delivery process. Curr. Drug Deliv..

[16] Nagy Z.K., Balogh A., Démuth B., Pataki H., Vigh T., Szabó B., Molnár K., Schmidt B.T., Horák P., Marosi G. (2015). High speed electrospinning for scaled-up production of amorphous solid dispersion of itraconazole. Int. J. Pharm..

[17] Wei L., Liu C., Mao X., Dong J., Fan W., Zhi C., Qin X., Sun R. (2019). Multiple-jet needleless electrospinning approach via a linear flume spinneret. Polymers.

[18] Qiu Q., Chen S., Li Y., Yang Y., Zhang H., Quan Z., Qin X., Wang R., Yu J. (2020). Functional nanofibers embedded into textiles for durable antibacterial properties. Chem. Eng. J..

[19] Chen C.-Y., Huang S.Y., Wan H.-Y., Chen Y.-T., Yu S.-K., Wu H.-C., Yang T.-I. (2020). Electrospun hydrophobic polyaniline/silk fibroin electrochromic nanofibers with low electrical resistance. Polymers.

[20] Smith S., Delaney M., Frey M. (2020). Anti-escherichia coli functionalized silver-doped carbon nanofibers for capture of E. coli in microfluidic systems. Polymers.

[21] Ding Y., Dou C., Chang S., Xie Z., Yu D.-G., Liu Y., Shao J. (2020). Core-shell Eudragit S100 nanofibers prepared via triaxial electrospinning to provide a colon-targeted extended drug release. Polymers.

[22] George M.C., Braun P.V. (2009). Multicompartmental materials by electrohydrodynamic cojetting. Angew. Chem. Int. Ed..

[23] Xiao Q., Lim L.-T. (2018). Pullulan-alginate fibers produced using free surface electrospinning. Int. J. Biol. Macromol..

[24] Wang M., Yu D.-G., Li X., Williams G.R. (2020). The development and bio-applications of multifluid electrospinning. Mater. Highlights.

[25] Huang C.-K., Zhang K., Gong Q., Yu D.-G., Wang J., Tan X., Quan H. (2020). Ethylcellulose-based drug nano depots fabricated using a modified triaxial electrospinning. Int. J. Biol. Macromol..

[26] Hou J., Yang J., Zheng X., Wang M., Liu Y., Yu D.-G. (2020). A nanofiber-based drug depot with high drug loading for sustained release. Int. J. Pharm..

[27] Wang K., Wang P., Wang M., Yu D.-G., Wan F., Bligh S.W.A. (2020). Comparative study of electrospun crystal-based and composite-based drug nano depots. Mater. Sci. Eng. C..

[28] Yu D.-G., Wang M., Li X., Liu X., Zhu L.-M., Annie Bligh S.W. (2020). Multifluid electrospinning for the generation of complex nanostructures. Wiley Interdiscip. Rev. Nanomed. Nanobiotechnol..

[29] Wang K., Wen H.-F., Yu D.-G., Yang Y., Zhang D.-F. (2018). Electrosprayed hydrophilic nanocomposites coated with shellac for colon-specific delayed drug delivery. Mater. Design.

[30] Nguyen J., Stwodah R.M., Vasey C.L., Rabatin B.E., Atherton B., D’Angelo P.A., Swana K.W., Tang C. (2020). Thermochromic fibers via electrospinning. Polymers.

[31] Singh A., Rath G., Singh R., Goyal A.K. (2018). Nanofibers: An effective tool for controlled and sustained drug delivery. Curr. Drug Deliv..

[32] Zhi L., Lei Z., Fangtao R., Anfang W., Jianghui Z., Quan F. (2020). Needle-disk electrospinning: Mechanism elucidation, parameter optimization and productivity improvement. Recent Pat. Nanotechnol..

[33] Zhao J., Li X., Liu Z. (2019). Needle’s vibration in needle-disk electrospinning process: Theoretical model and experimental verification. J. Low Freq. Noise V. A..

[34] Vass P., Pantea E., Domokos A., Hirsch E., Domján J., Németh Á., Molnár M., Fehér C., Andersen S.K., Vigh T. (2020). Electrospun Solid Formulation of Anaerobic Gut Microbiome Bacteria. AAPS PharmSciTech.

[35] Yildiz Z.I., Celebioglu A., Uyar T. (2017). Polymer-free electrospun nanofibers from sulfobutyl ether7-beta-cyclodextrin (SBE7-β-CD) inclusion complex with sulfisoxazole: Fast-dissolving and enhanced water-solubility of sulfisoxazole. Int. J. Pharm..

[36] Chang S., Wang M., Zhang F., Liu Y., Liu X., Yu D.-G., Shen H. (2020). Sheath-separate-core nanocomposites fabricated using a trifluid electrospinning. Mater. Design.

[37] Yang Y., Chang S., Bai Y., Du Y., Yu D.-G. (2020). Electrospun triaxial nanofibers with middle blank cellulose acetate layers for accurate dual-stage drug release. Carbohydr. Polym..

[38] Chen S., Xie Y., Chinnappan A., Wei Z., Gu Q., He H., Fang Y., Zhang X., Lakshminarayanan R., Zhao W. (2020). A self-cleaning zwitterionic nanofibrous membrane for highly efficient oil-in-water separation. Sci. Total Environ..

[39] Farokhi M., Mottaghitalab F., Reis R.L., Ramakrishna S., Kundu S.C. (2020). Functionalized silk fibroin nanofibers as drug carriers: Advantages and challenges. J. Control. Release.

[40] Celebioglu A., Uyar T. (2020). Fast-dissolving antioxidant curcumin/cyclodextrin inclusion complex electrospun nanofibrous webs. Food Chem..

[41] Vass P., Démuth B., Farkas A., Hirsch E., Szabó E., Nagy B., Andersen S.K., Vigh T., Verreck G., Csontos I. (2019). Continuous alternative to freeze drying: Manufacturing of cyclodextrin-based reconstitution powder from aqueous solution using scaled-up electrospinning. J. Control. Release.

[42] Celebioglu A., Kayaci-Senirmak F., İpek S., Durgun E., Uyar T. (2016). Polymer-free nanofibers from vanillin/cyclodextrin inclusion complexes: High thermal stability, enhanced solubility and antioxidant property. Food Funct..

[43] Vass P., Szabó E., Domokos A., Hirsch E., Galata D., Farkas B., Démuth B., Andersen S.K., Vigh T., Verreck G. (2020). Scale-up of electrospinning technology: Applications in the pharmaceutical industry. Wiley Interdiscip. Rev. Nanomed. Nanobiotechnol..

[44] Yang Y., Zhao Y., Quan Z., Zhang H., Qin X., Wang R., Yu J. (2019). An efficient hybrid strategy for composite yarns of micro-/nano-fibers. Mater. Design.

[45] Zhang H., Ye J., Qin X. (2019). Facile fabrication and transistor properties of mixed crystalline TiO_2_ nanofibers FET devices. Mater. Lett..

[46] Kim M.-W., An S., Seok H., Jung H., Park D.-H., Yarin A.L., Yoon S.S. (2020). In vitro evaluation of Pt-coated electrospun nanofibers for endovascular coil embolization. Acta Biomater..

[47] Wang P., Wang M.L., Wan X., Zhou H., Zhang H., Yu D.G. (2020). Dual-stage release of ketoprofen from electrosprayed core-shell hybrid polyvinyl pyrrolidone/ethyl cellolose nanoparticles. Mater. Highlights.

[48] Bai Y., Wang D., Zhang Z., Pan J., Cui Z., Yu D.G., Annie Bligh S.W. (2020). Testing of fast dissolution of ibuprofen from its electrospun hydrophilic polymer nanocomposites. Polym. Test..

[49] Yu D.G., Gao L.D., White K., Brandford-White C., Lu W.Y., Zhu L.M. (2010). Multicomponent amorphous nanofibers electrospun from hot aqueous solutions of a poorly soluble drug. Pharm. Res..

[50] Zheng G., Jiang J., Wang X., Li W., Zhong W., Guo S. (2018). Self-cleaning threaded rod spinneret for high-efficiency needleless electrospinning. Appl. Phys. A.

[51] Liu Z., Chen R., He J. (2016). Active generation of multiple jets for producing nanofibres with high quality and high throughput. Mater. Design.

[52] Xu F., Gough I., Dorogin J., Sheardown H., Hoare T. (2020). Nanostructured degradable macroporous hydrogel scaffolds with controllable internal morphologies via reactive electrospinning. Acta Biomater..

[53] Xu F., Dodd M., Sheardown H., Hoare T. (2018). Single-step reactive electrospinning of cell-loaded nanofibrous scaffolds as ready-to-use tissue patches. Biomacromolecules.

[54] Sivakumaran D., Bakaic E., Campbell S.B., Xu F., Mueller E., Hoare T. (2018). Fabricating degradable thermoresponsive hydrogels on multiple length scales via reactive extrusion, microfluidics, self-assembly, and electrospinning. JoVE.

[55] Vass P., Nagy Z.K., Kóczián R., Fehér C., Démuth B., Szabó E., Andersen S.K., Vigh T., Verreck G., Csontos I. (2020). Continuous drying of a protein-type drug using scaled-up fiber formation with HP-β-CD matrix resulting in a directly compressible powder for tableting. Eur. J. Pharm. Sci..

[56] Vass P., Démuth B., Hirsch E., Nagy B., Andersen S.K., Vigh T., Verreck G., Csontos I., Nagy Z.K., Marosi G. (2019). Drying technology strategies for colon-targeted oral delivery of biopharmaceuticals. J. Control. Release.

[57] Costa P., Sousa Lobo J.M. (2001). Modeling and comparison of dissolution profiles. Eur. J. Pharm. Sci..

[58] Korsmeyer R.W., Gurny R., Doelker E., Buri P., Peppas N.A. (1983). Mechanisms of solute release from porous hydrophilic polymers. Int. J. Pharm..

[59] Vithani K., Jannin V., Pouton C.W., Boyd B.J. (2019). Colloidal aspects of dispersion and digestion of self-dispersing lipid-based formulations for poorly water-soluble drugs. Adv. Drug Deliv. Rev..

[60] Nagy Z.K., Balogh A., Vajna B., Farkas A., Patyi G., Kramarics Á., Marosi G. (2012). Comparison of electrospun and extruded Soluplus^®^-based solid dosage forms of improved dissolution. J. Pharm. Sci..

[61] Junqueira L.A., Polonini H., Loures S., Raposo N.R.B., Ferreira A.O., Brandao M.A.F. (2019). Permeation efficacy of a transdermal vehicle with steroidal hormones and nonsteroidal anti-inflammatory agents as model drugs. Curr. Drug Deliv..

[62] Musazzi U.M., Selmin F., Ortenzi M.A., Mohammed G.K., Franzé S., Minghetti P., Cilurzo F. (2018). Personalized orodispersible films by hot melt ram extrusion 3D printing. Int. J. Pharm..

[63] Cilurzo F., Musazzi U.M., Franzé S., Selmin F., Minghetti P. (2018). Orodispersible dosage forms: Biopharmaceutical improvements and regulatory requirements. Drug Discov. Today.

[64] Musazzi U.M., Khalid G.M., Selmin F., Minghetti P., Cilurzo F. (2020). Trends in the production methods of orodispersible films. Int. J. Pharm..

[65] Thakkar S., More N., Sharma D., Kapusetti G., Kalia K., Misra M. (2019). Fast dissolving electrospun polymeric films of anti-diabetic drug repaglinide: Formulation and evaluation. Drug Dev. Ind. Pharm..

[66] Mofidfar M., Prausnitz M.R. (2019). Electrospun transdermal patch for contraceptive hormone delivery. Curr. Drug Deliv..

[67] Torres-Martinez E.J., Cornejo Bravo J.M., Serrano Medina A., Pérez González G.L., Villarreal Gómez L.J. (2018). A summary of electrospun nanofibers as drug delivery system: Drugs loaded and biopolymers used as matrices. Curr. Drug Deliv..

[68] Al-Mezrakchi R.Y.H., Naraghi M. (2018). Interfused nanofibres network in scalable manufacturing of polymeric fibres via multi-nozzle electrospinning. Micro Nano Lett..

[69] Ramakrishnan R., Gimbun J., Ramakrishnan P., Ranganathan B., Reddy S.M.M., Shanmugam G. (2019). Effect of solution properties and operating parameters on needleless electrospinning of poly (ethylene oxide) nanofibers loaded with bovine serum albumin. Curr. Drug Deliv..

[70] He J.X., Qi K., Wang L.D., Zhou Y.M., Liu R.T., Cui S.Z. (2015). Combined application of multinozzle air-jet electrospinning and airflow twisting for the efficient preparation of continuous-twisted nanofiber yarn. Fiber. Polym..

