# Correction: Kang et al. Energy-Saving Electrospinning with a Concentric Teflon-Core Rod Spinneret to Create Medicated Nanofibers. *Polymers* 2020, *12*, 2421

**DOI:** 10.3390/polym18121451

**Published:** 2026-06-10

**Authors:** Shixiong Kang, Shicong Hou, Xunwei Chen, Deng-Guang Yu, Lin Wang, Xiaoyan Li, Gareth R. Williams

**Affiliations:** 1 School of Materials Science & Engineering, University of Shanghai for Science & Technology, 516 Jungong Road, Shanghai 200093, China; 182442511@st.usst.edu.cn (S.K.); 183762605@st.usst.edu.cn (S.H.); 1826418107@st.usst.edu.cn (X.C.); lixiaoyan@usst.edu.cn (X.L.); 2 Shanghai Institute of Technical Physics, Chinese Academy of Sciences, 500 Yutian Road, Shanghai 200083, China; wanglin@mail.sitp.ac.cn; 3 UCL School of Pharmacy, University College London, 29-39 Brunswick Square, London WC1N 1AX, UK

In the original publication [1], there was a mistake in Figure 8 as published. There was no Y-axis for the XRD patterns and FTIR spectra. We have redone the experiments and converted the data in a better manner. The corrected Figure 8 appears below, which is based on newly repeated experiments. 

The reasons for the previous lack of a Y-axis for XRD patterns have been explained in another correction of [2]. We repeat them as follows: (1) Here, XRD and FTIR are qualitative analyses; (2) the only meaning of XRD patterns is to disclose the physical state of drug molecules in the electrospun nanofibers by the presence or absence of sharp Bragg peaks [3,4,5,6,7,8], and the only meaning of FTIR spectra is to disclose the compatibility between KET and PVP; (3) sampling is not identical for all the samples; (4) the physical quantity of the Y-axis is unit-less intensity, and its values and related noises have no scientific meaning, suggest nothing, form no judgments, and have no influence on the conclusions; (5) it is often very hard to include all XRD patterns into one figure using the same scale range, or in doing so some XRD patterns become straight smooth lines; and (6) numerous publications have no Y-axes for their XRD patterns and also no FTIR spectra [9,10,11]. However, the presence of a Y-axis may make the readings of XRD patterns and FTIR spectra easier for the readers. 

The authors state that the scientific conclusions are unaffected. This correction was approved by the Academic Editor. The original publication has also been updated.

## Figures and Tables

**Figure 8:**
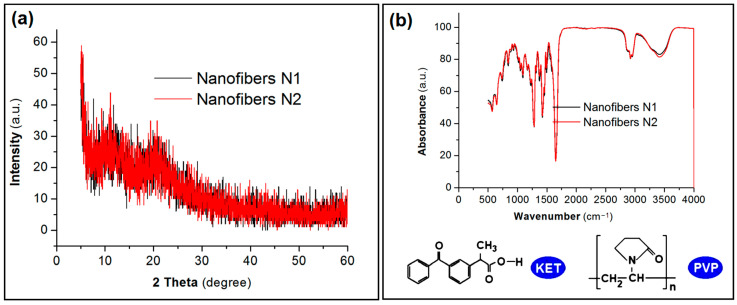
XRD patterns (**a**) and ATR-FTIR spectra (**b**) of nanofibers N1 and N2.
