# Supplementary material for: Energy-Saving Electrospinning with a Concentric Teflon-Core Rod Spinneret to Create Medicated Nanofibers
Source: Polymers (Basel). 2020 Oct 20;12(10):2421. doi: 10.3390/polym12102421 (PMC7589577; doi:10.3390/polym12102421)
Supplement: Supplementary file 1 [file polymers-12-02421-s001.pdf]

Supplementary Information

Energy-saving electrospinning with a concentric Teflon-core rod spinneret to create medicated nanofibers

Shixiong Kang,[1, a] Shicong Hou,[1, 2, a] Xunwei Chen,[1] Deng-Guang Yu,[1,*]
Lin Wang,[2] Xiaoyan Li,[1] Gareth R. Williams [3,*]

[1] School of Materials Science & Engineering, University of Shanghai for Science and Technology, Shanghai 200093, China
[2] Shanghai Institute of Technical Physics, Chinese Academy of Sciences, 500 Yutian Road, Shanghai 200083, China
[3] UCL School of Pharmacy, University College London, 29-39 Brunswick Square, London WC1N 1AX, UK

[a] These authors contributed equally to this work.

*** Corresponding authors:**
Prof. Deng-Guang Yu
School of Materials Science & Engineering
University of Shanghai for Science and Technology
Address: 516 Jungong Road, Yangpu District, Shanghai 200093, China
E-mail: ydg017@usst.edu.cn
Tel/Fax: +86-21-55270632

Prof. Gareth R. Williams
UCL School of Pharmacy, University College London
29-39 Brunswick Square, London WC1N 1AX, UK
E-mail: g.williams@ucl.ac.uk

S1. Experimental

S1-1. *Observations of the new spinneret for conducting different sorts of electrohydrodynamic atomization (EHDA) processes*

A series of working fluids with different concentrations of KET and PVP were prepared according to Table S1. These working fluids were all subjected to the EHDA processes using the electrospinning system with the concentric spinneret with a core solid Teflon needle.

Table S1 The parameters for EHDA processes on different working fluids

No.	Preparation of working fluids			Working conditions		
	KET (g)	PVP (g)	Ethanol (mL)	Concentration of PVP (w/v)%	Applied voltage (kV)	Collected distance (cm)
E1	1	4	10	4	15	15
E2	1.5	6	10	6	12	20
N2	10	40	500	8	12	20
E3	2.5	10	10	10	12	20
E4	3	12	10	12	12	20
E5	3.75	15	10	15	12	20

A 59XA-2 polarized microscope (Shanghai Optical Instrument Factory, Shanghai, China) was used to observe the collected EHDA products at a magnification of 20×40.

S1-2. *Observations of the fast dissolution of nanofibers N1 and N2 through the Drop Shape Analysis experiments*

A drop shape analysis instrument (DSA100, Kruss GmbH, Hamburg, Germany) is exploited to investigate the fast dispersible properties of the electrospun nanofibers N1 and N2, which were produced using different spinnerets. A volume of about 3 µm doubly distilled water was dropped on the surface of a nanofiber film. The receded processes of a water droplet were recorded, which can be reflected by the surface water contact angle (WCA).

S2. Results and discussion

S2-1. *Observations of the new spinneret for conducting different sorts of electrohydrodynamic atomization (EHDA) processes*

As anticipated, the concentric spinneret with a core solid Teflon rod can be explored for creating different sorts of EHDA products, which varied from small particles (Fig. S1a), beads-on-a-string hybrids (Fig. S1b), linear nanofibers with different diameters (Fig. S1c to S1e), and adherent fibers (Fig. S1f), for EHDA products E1, E2, N2, E3, E4, and E5, respectively. These results suggest that the new spinneret can be exploited for conducting all types of EHDA processes in an energy-saving manner.

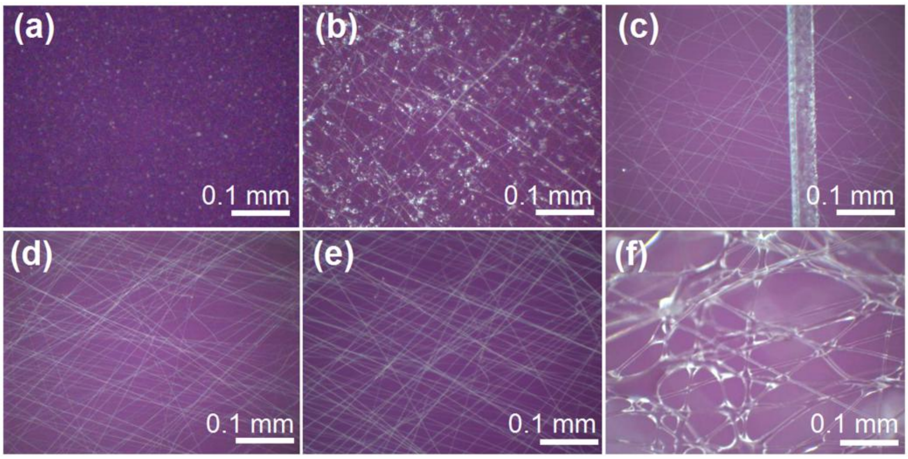

Fig. S1. Polarized microscopic images of the EHDA products from working fluids with varied PVP and KET concentrations, which were created using the coaxial spinneret with a core solid Teflon rod: (a) E1; (b) E2; (c) N2; (d) E3; (e) E4; (f) E5.

S2-2. *Observations of the fast dissolution of nanofibers N1 and N2 through the Drop Shape Analysis experiments*

When a drop of water was placed on the surface of nanofibers N1 from the tube-based electrospinning, the droplet quickly disappeared from the view. The whole process is included in **Fig. S2.** The surface was flattened again in 4.86±0.63 minutes (n=6).

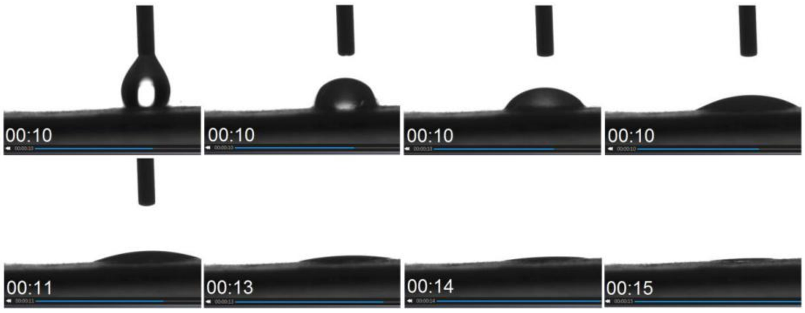

Fig. S2. Observations of the fast disappearance of a drop of water when it is placed on the surface of electrospun nanofibers N1.

Similarly, when a drop of water was placed on the surface of nanofibers N2 from the new electrospinning using the concentric spinneret with a core solid Teflon rod, the droplet disappeared from the view rapidly. The whole process is included in **Fig. S3.** The surface was flattened again in 4.71±0.69 minutes (n=6).

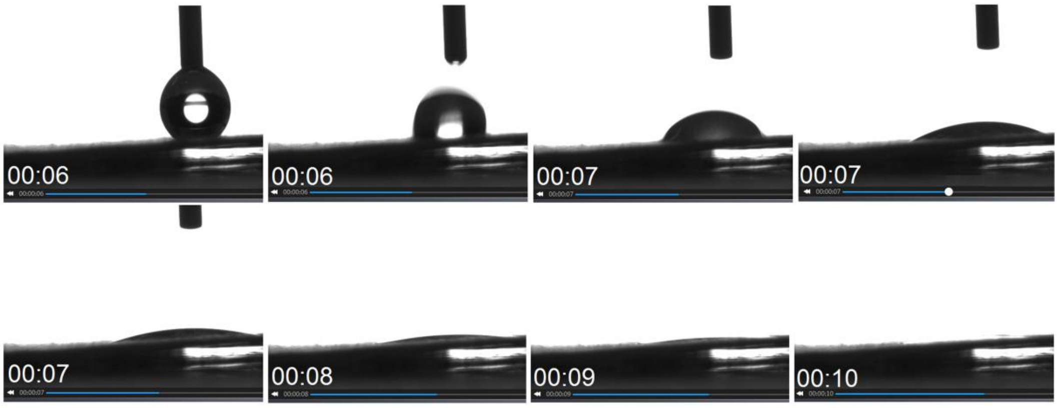

Fig. S3. Observations of the fast disappearance of a drop of water when it is placed on the surface of electrospun nanofibers N2.
